# Daily fluctuations in sleep duration and quality affect next-day processing speed performance in young and older adults: an intensive longitudinal everyday life study over 21 days

**DOI:** 10.1093/sleep/zsaf321

**Published:** 2025-10-14

**Authors:** Johanna Schwarz, Malin Freidle, Wessel van Leeuwen, Jade Silfverling, Torbjörn Åkerstedt, Göran Kecklund

**Affiliations:** Department of Psychology, Stockholm University, Stockholm, Sweden; Department of Clinical Neuroscience, Karolinska Institute, Stockholm, Sweden; Department of Psychology, Stockholm University, Stockholm, Sweden; Department of Psychology, Stockholm University, Stockholm, Sweden; Department of Psychology, Stockholm University, Stockholm, Sweden; Department of Psychology, Stockholm University, Stockholm, Sweden; Department of Clinical Neuroscience, Karolinska Institute, Stockholm, Sweden; Department of Psychology, Stockholm University, Stockholm, Sweden

**Keywords:** actigraphy, aging, cognitive function, neurobehavioral performance, ecological momentary assessment (EMA), experience sampling method (ESM), smartphone-based cognitive testing, remote cognitive testing, daily life, sleep

## Abstract

**Study Objectives:**

Knowledge about how day-to-day variations in sleep affect cognitive performance in real-world contexts is currently limited. This study investigated how daily fluctuations in sleep duration, efficiency, and quality affect next-day processing speed, and tested whether these associations differ between young and older adults.

**Methods:**

A total of 158 young (18–30 years) and 168 older adults (55–75 years) participated in a 21-day intensive longitudinal design. Sleep duration and efficiency were measured using actigraphy, while sleep quality was assessed via sleep diaries. Processing speed was measured using a 60 s smartphone-based Digit Symbol Substitution Task, administered up to eight times per day. Multilevel mixed models tested the within- and between-person effects of sleep duration, sleep efficiency and sleep quality, as well as the effect of age group on processing speed.

**Results:**

Within-person, a sleep duration shorter than their own average (*p* < .001), and a sleep quality poorer than their own average (*p* < .05) predicted poorer next-day performance. Between-person differences in sleep duration, sleep efficiency and sleep quality were not significantly associated with processing speed. Older adults showed worse performance than young adults (*p* < .001), but the effect of daily sleep fluctuations on performance did not significantly vary between age groups.

**Conclusions:**

Daily fluctuations in sleep duration and sleep quality are linked to processing speed in young and older adults in real-world contexts. Results suggest that within-person, day-to-day variations in sleep may be more important than between-person differences. Maintaining an adequate sleep duration each day may help prevent cognitive impairments in daily functioning across age groups.

## Teaser

**Statement of Significance** This study examined how daily fluctuations in sleep duration, sleep efficiency, and sleep quality influence processing speed in real-world settings, using a large sample of young and older adults. The results show that within-person day-to-day changes, but not between-person differences, in objectively measured sleep duration and subjective sleep quality are associated with next-day processing speed performance. These findings highlight the importance of getting adequate sleep each day to support cognitive functioning in everyday life for both young and older adults.

## Graphical Abstract

**Graphical Abstract:**
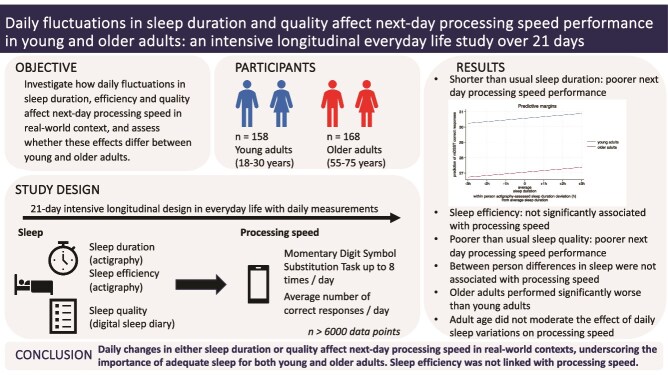

## Introduction

The importance of sleep for cognition is well-established, with extensive laboratory research demonstrating that both total and partial sleep deprivation impair cognitive performance [1–3]. Of note, the effects of experimental sleep loss appear to vary across the adult lifespan, with young adults being more sensitive to sleep deprivation compared to older adults [4, 5]. While these experimental studies have established a causal relationship between sleep loss and cognitive functioning, they have limited ecological validity. Day-to-day variations in sleep are very common [6, 7], yet there is only scarce knowledge on how these within-person fluctuations in sleep and sleep quality influence cognitive performance in the natural daily environment. Therefore, this study aims to investigate how daily changes in sleep across 21 days are associated with next-day cognitive performance in a processing speed task in young and older adults in the context of everyday life.

Processing speed, the ability to quickly and accurately evaluate information and respond accordingly [8], is an important cognitive capability. Deficits in processing speed are thought to be linked to impairment in both simple and complex cognitive processes [9], and are considered central for the impairment in a wide range of age-related cognitive processes [10]. Experimental studies have shown that both total [11, 12] and partial sleep deprivation [13, 14] cause decrements in performance on the Digit Symbol Substitution Task (DSST), a frequently used task to measure processing speed, which was also used in the present study. Moreover, a large cross-sectional study involving around 500 000 individuals aged 15–89 years found a small inverted U-shaped association between self-reported sleep duration and processing speed, with both short and long sleep associated with poorer performance [15]. While a meta-analysis focusing on older adults found no significant association between objectively measured sleep parameters, including sleep duration and efficiency, and processing speed [16], a more recent study reported that lower sleep efficiency was linked to poorer DSST performance [17].

Although laboratory-based sleep deprivation studies are essential to test the causal impact of sleep loss on cognitive abilities such as processing speed, it is unclear how these findings translate to naturally occurring sleep variations in real world contexts. While sleep commonly varies from day to day [6, 7], the sleep loss experienced in everyday life is presumably less extreme than the sleep deprivation in experimental studies, which commonly involves either a complete night without sleep or severe sleep restrictions to 4 h or less for one or more nights. Thus, its impact may differ and be more easily compensated for. Furthermore, laboratory experiments usually include highly selected healthy participants who may not be representative of the general population, particularly so in older adults, where sleep disturbances [18] and health issues are more prevalent. This limitation is especially noteworthy given findings that suggest that older adults, despite age-related declines in sleep duration, and changes in sleep architecture and microstructure [19, 20], appear to be less vulnerable to the effects of sleep loss compared to young adults. Specifically, experimental sleep deprivation studies have shown that older adults show less performance degradation in sustained attention tasks [4, 5, 21], and fewer impairments in driving performance [22], while performance in a more complex cognitive task was similarly affected in both young and older adults [21]. In addition, it needs to be taken into account that laboratory-based cognitive testing sets out to measure optimal cognition under standardized and artificial conditions, while typical cognitive functioning in daily life is influenced by contextual factors and distractions that the individual has to navigate [23].

Therefore, there is now also growing interest in measuring cognitive performance, and variability thereof, in the context of everyday life. However, to date there is only very limited knowledge on how daily variations in sleep affect cognitive performance in real-world settings. In elementary school children, sleep quality and time in bed predicted performance in a working memory task in the morning, but not in the afternoon [24]. In adolescents, shorter sleep duration and poorer sleep efficiency were associated with a small decrease in performance in the Psychomotor Vigilance Task the next day [25]. In older adults, Lucke, Wrzus, Gerstorf, Kunzmann, Katzorreck, Schmiedek, Hoppmann, and Schilling [26] found an association between day-to-day changes in self-reported sleep duration and working memory performance in individuals who slept relatively little on average. Yet, it remains unclear whether these findings extend to different cognitive domains. Moreover, no study to date has directly compared young and older adults, and it remains unknown whether the lower susceptibility to sleep loss found in experimental studies [4, 27], also applies to natural variations in sleep in real-world settings.

Therefore, the main aim of this study was to examine the association of day-to-day changes in sleep on processing speed in the context of real life across 21 days. Sleep was assessed using both actigraphically measured sleep duration and efficiency, to capture sleep quantity and continuity as important aspects of objective sleep quality, as well as self-reports of sleep quality as predictors. Moreover, we wanted to investigate, whether there are differences between young and older adults. As outlined in the study protocol [28], we hypothesized the following regarding the relationship between daily sleep variations and cognitive performance in terms of processing speed:

*H1.1a*: Shorter sleep duration than usual (i.e. shorter sleep duration than a person’s own average) is associated to worse cognitive performance.

*H1.1b*: This association between sleep duration and cognitive performance is less strong in older adults than in young adults.

*H1.2a*: Lower sleep efficiency than usual (i.e. lower sleep efficiency than a person’s own average) is associated to worse cognitive performance.

*H1.2b*: This association between sleep efficiency and cognitive performance is less strong in older adults than in young adults.

*H1.3a*: Poorer self-reported sleep quality than usual (i.e. poorer sleep quality than a person’s own average) is associated to worse cognitive performance.

*H1.3b*: This association between self-reported sleep quality and performance is different in older adults than in young adults.

## Methods

### Participants

This study uses data from 158 participants aged 18–30 years (“young adults”) and 168 participants aged 55–75 years, a group spanning late midlife to older adulthood and referred to here as “older adults,” who took part in the *Sleep in Everyday Life* study [28].

Table 1 gives an overview of descriptive sociodemographic variables for the participants. An a priori simulation-based power calculation [28] determined that 160 participants per age group would ensure adequate statistical power for detecting the expected interaction effect between age group and within-person deviations of sleep duration on processing speed.

**Table 1:** Descriptive results for sociodemographic variables. Values are presented as *N* or mean (standard deviation)

	**Young adults**	**Older adults**
*N*	158	168
Sex		
Women	81	98
Men	77	70
Age (years)	23.76 (3.00)	63.55 (5.67)
Body mass index (kg/m^2^)^a^	23.50 (3.21)	26.15 (3.68)
Education level		
Below bachelor degree	104	77
Bachelor degree or higher	54	91
Occupation		
Studying	136	10
Working full-time	24	63
Working part-time	31	28
Unemployed	6	6
Position of trust	1	10
Volunteering	5	23
Part-time retired	0	6
Full-time retired	0	71
Mini Mental State Examination (points)	28.71 (1.29)	28.73 (1.54)

Occupational status was assessed via multiple choice.

a *n* = 1 missing.

Inclusion criteria for the study comprised age criteria (18–30 years for the young adult group, 55–75 years for the older adult age group), having a Swedish social security number, being fluent in Swedish, being able to travel on-site twice for the briefing and debriefing meeting, having no indication of cognitive impairment (Mini Mental State Examination > 24) and to have a smartphone that was compatible with the mobile application used in this study. Participants had also to be able to wear an actigraph 24/7. Exclusion criteria comprised health conditions and/or medical treatments that likely impact daily physical activity, sleep, psychological well-being or performance ability to a great extent. Exclusion decisions were made on a case-by-case basis. Examples of health conditions and treatments (self-reported) deemed to warrant exclusion included a history of stroke, dementia, or Parkinson’s disease, as well as a recent (within the past 6 months) diagnosis or treatment for exhaustion disorder, depression, anxiety disorders, cancer, or the use of antidepressants. Individuals with sleep disorders or recent treatment (within the past 6 months) for sleep disorders—including sleep apnea, insomnia, and narcolepsy—with the exception of restless legs syndrome, were excluded. Additional exclusion criteria included an Insomnia Severity Index score greater than 21 and/or the use of sleep medication more than once per week. Further exclusion criteria were lack of ability or confidence to use a smartphone, functional damage of the smartphone, work requiring wakefulness between 2:00 and 5:00 once a month or more often (exception of on-call work), and inability to establish contact with participants. The complete study and enrolment procedure is described in more detail in the study protocol [28]. In addition to the participants excluded due to inclusion and exclusion criteria, six participants were excluded after the data collection (two young adults due to aborting the data collection, two young adults and one older adult due to very irregular and difficult-to-score sleep–wake patterns, and one young adult due to severe personal events during the study period).

For the present analyses, which focus on how day-to-day deviations from usual sleep patterns impact next-day processing speed, we also excluded participants with fewer than 10 nights of actigraphically recorded sleep (*n* = 6 young adults, *n* = 5 older adults) or fewer than 10 nights with sleep quality ratings (*n* = 4 young adults, *n* = 3 older adults) paired with cognitive performance tests the following day. This threshold aligns with findings by Lau et al. (2022) reporting that 10 nights render a very good estimate for monthly sleep duration. Moreover, one participant (older adult group) was excluded because the response pattern indicated pronounced difficulties in completing the processing speed test (>50% occasions with 0 correct responses).

The study was approved by the Swedish Ethical Review Authority, and all participants signed written informed consent. Participants were economically compensated for their participation using a staggered payment scheme depending on the number of daily questionnaires they filled in. Participants received a maximum daily compensation (approximately 15 USD) for completing 7–10 questionnaires, an intermediate amount (approximately 7.5 USD) for 4–6, and a low amount (approximately 1.5 USD) for 0–3. The maximum total possible compensation for participating in the study was 310 USD (approximately) before tax.

### Study design

In this intensive longitudinal study, young (18–30 years) and older (55–75 years) participants completed daily questionnaires and processing speed tests via their mobile phones over a 21-day period, while wearing actigraphs to objectively measure sleep.

### Procedure

After passing an initial online screening form addressing questionnaire-based inclusion and exclusion criteria, participants attended an on-site briefing session, where the experience sampling application PsyMate^TM^ (www.psymate.eu) was installed on their own smartphone. During the briefing session, they were familiarized with how to operate the application, respond to the questionnaire prompts and completed the cognitive task (momentary Digit Symbol Substitution Task [mDSST]), see Schwarz, Freidle, van Leeuwen, Åkerstedt, and Kecklund [28] for more details. Due to technical problems with the installation, eight participants installed the application remotely but were trained on site and received written instructions by e-mail. Participants received a scheduled phone call about 2–3 days after starting the data collection by a study assistant to check in with the participants. They also had the possibility to reach out to the study assistants throughout the data collection in case of questions.

During the 21-day data collection, participants were asked to continuously wear an actigraph (Motionwatch 8 by CamNtech, Cambridge, UK) on their non-dominant wrist, and to only take it off when immersing the wrists for a longer time in water, e.g. when going swimming. Every day, participants were asked to complete a number of questionnaires via the PsyMate^TM^ app on their smartphone. As part of the morning questionnaire (available between 05:00 and 13:00), they answered questions on the quality of the previous night’s sleep. At eight times during the day, participants received prompts to answer daytime questionnaires, which included the 60 s processing speed task (mDSST). The daytime questionnaires were scheduled pseudo-randomly within 2-h intervals between 07:00 and 23:00, ensuring that they were at least 30 min apart from each other. They expired after 20 min in order to ensure that responses reflected in the moment experiences. Before going to bed, participants were in addition asked to complete an evening questionnaire, which was available between 19:00 and 04:00. Data collection took place between October 2022 and October 2023.

## Measures

### Sleep duration and efficiency

Objective estimates of sleep duration and sleep efficiency for the night’s sleep were determined at the 30-s epoch level using the Motionwatch 8 (CamNtech, Cambridge, UK), an actigraphy device with good validity against polysomnography estimates [29]. Sleep was scored using the built-in auto-sleep detection from the MotionWare software (CamNtech, Cambridge, UK). The auto-sleep configuration was set to a sleep activity threshold of 20 cpm (default), a minimum sleep fraction of 60% (default), a prescan sleep fraction of 80% (default), and a marker button limit of 0 h. Hence, marker button presses had, with the current settings, no effect on the auto-sleep scorings.

Auto-sleep scorings were adjusted based on predefined and preregistered criteria (https://osf.io/7nzgw/). Briefly, auto-sleep was not adjusted if other indicators (i.e. marker button press and/or sleep diary) of sleep on- or offset were within 15 min from the auto-sleep. In case longer distances were observed between the different indicators (i.e. auto-sleep, marker button press, and sleep diary), significant changes in activity were taken as sleep on- or offset, provided that either a marker button press or a sleep diary entry was within a 15-min range of such a significant change. If not even that was the case, auto-sleep was manually judged based on overall recorded activity and light levels. In the latter case, a second actigraphy expert was also consulted for confirmation. For a sleep to be split into two parts (a so-called split-sleep), a period of wakefulness in between of at least 1 h was required.

Sleep duration was based on the actual sleep time output of the sleep analyses and expressing this duration as a percentage of time in bed would result in the sleep efficiency variable.

### Sleep quality

Subjective sleep quality was measured by aggregating answers from four items in the Karolinska Sleep Diary (sleep quality, calmness of sleep, ease of falling asleep, and sleep maintenance) to a sleep quality index [30]. Participants answered the items as part of the daily morning questionnaire. Higher values (ranging from 1 to 5) indicate poorer sleep quality.

### Processing speed

**Figure 1:**
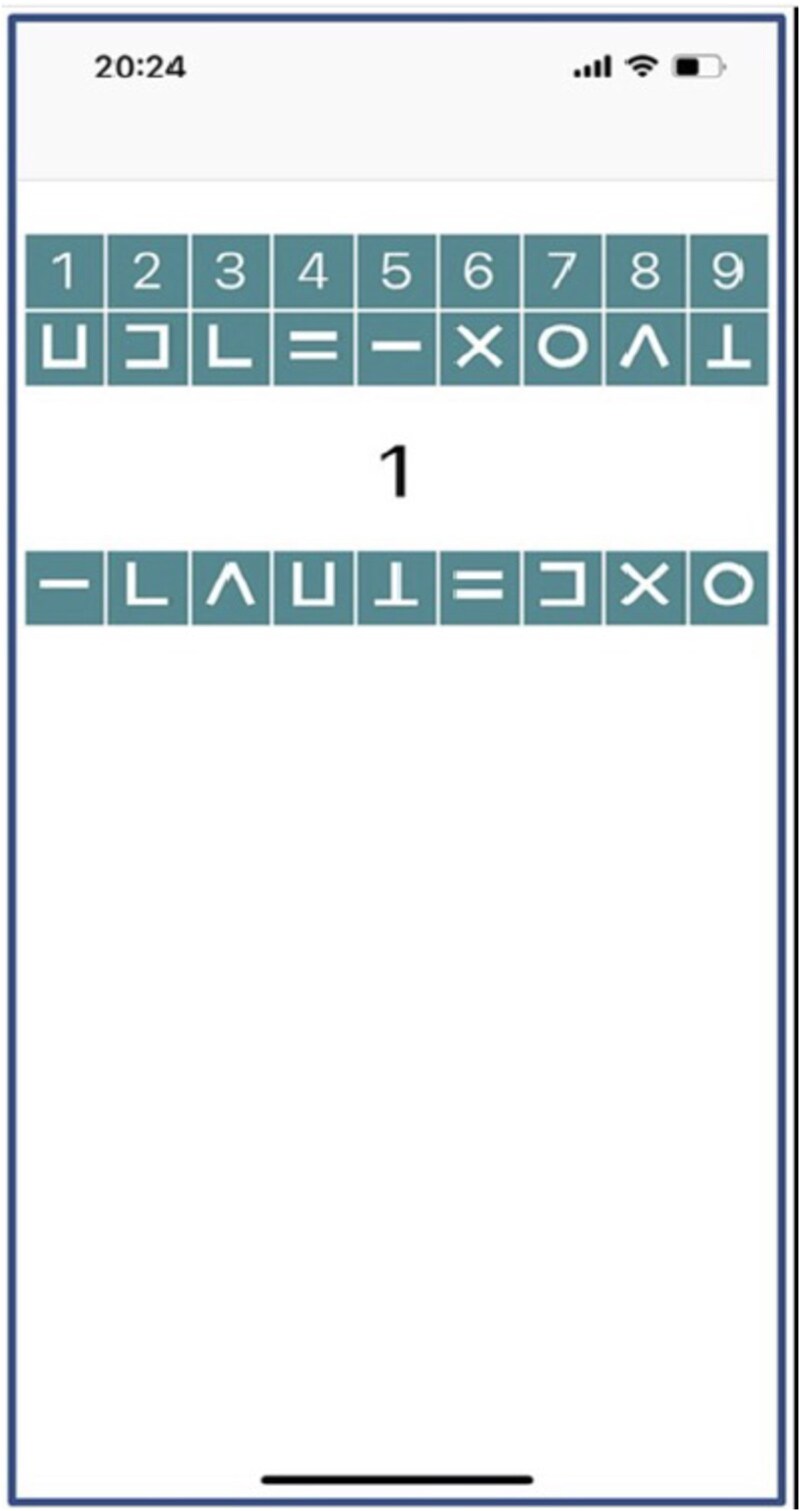
Illustration of a momentary Digit Symbol Substitution Task (mDSST) trial. In the mDSST, digits from 1 to 9 are displayed at the top of the screen, each paired with a unique symbol forming a reference key. On each trial, one digit is presented as a probe in the center of the screen, and participants are asked to select the matching symbol. Figure is reproduced from [28], under CC BY 4.0.

Processing speed was measured using the average number of correct responses per day in a 60 s version of the PsyMate^TM^ mDSST. Participants completed the mDSST up to eight times a day as part of the daytime questionnaires. Adapted from the DSST included in the Wechsler Adult Intelligence Scale, the mDSST has been successfully implemented in previous mobile experience sampling studies [31, 40]. In the mDSST, the digits 1–9 are presented on top of the screen, each combined with a symbol. On each trial, a digit is presented as a probe in the middle of the screen (see Figure 1). The participant’s task is to select the corresponding symbol at the bottom of the screen. The symbol-digit combinations were alternated for each test session, but stayed constant within each test session. Participants were instructed to complete as many trials as possible while also being as accurate as possible. Due to the short task duration, and the one-by-one presentation, this version of the mDSST primarily measures processing speed rather than working memory [31].

On average, young adults completed 5.04 mDSST sessions per day (SD = 1.13), older adults completed 5.95 mDSST sessions per day (SD = 1.14). Of note, 1.35% of the sessions were completed before the final wake up (according to actigraphy), which were included in the data analysis. To exclude extreme outliers, sessions with the number of correct responses exceeding ±3 standard deviations of the person mean were removed (1.15% of the sessions) before calculating the daily average of correct responses.

### Covariates

The following two time-invariant covariates were accounted for in the statistical analyses: (1) sex, which has been shown to affect processing speed [32] and sleep [33], (2) full-time retirement, which is negatively associated with processing speed [34] and positively with sleep [35]. Moreover, a linear and a quadratic term for study day were included as time-varying covariates to account for potential practice effects over the course of the study.

Since daily fluctuations in health and napping may also influence processing speed, we conducted a sensitivity analysis: Subjective health (categorical scale from 1 to 5) and self-reported napping (yes/no), as rated in the evening questionnaire, were included as time-varying covariates. Self-rated health, a well-established indicator of health risk, was chosen as it provides an inclusive measure that captures co-morbidities better compared to additive measures of diseases [36]. In addition, we included further covariates from the inclusion and background questionnaires in this sensitivity analysis: cohabitation status (married or living with partner vs. not married or living with partner), weekend/holiday vs. weekday, nicotine use (any reported current use of cigarettes, snus, or other nicotine products vs no current use), alcohol consumption (coded as 0 = no alcohol, 1 = one glass per week, and 2 = two or more glasses per week), and Insomnia Severity Index scores (continuous). The sensitivity analyses, which included fewer observations due to missing data for the covariates, yielded overall the same pattern of results for the main predictors. The results of the sensitivity analyses are included in Supplementary Materials S3–S5).

### Statistical analyses

To investigate whether daily changes in sleep, the between group factor adult age group, and their interaction are associated with next-day processing speed, multilevel linear mixed models were fitted using the STATA procedure mixed, with the average number of correct trials as the outcome measure, as planned in the study protocol [28]. The analyses included all available data, assuming that missing values were missing at random.

We fitted separate models for each sleep predictor (sleep duration, sleep efficiency, and sleep quality). In order to disaggregate between- and within-person effects, the time-varying sleep predictors were person-mean centered [37, 38], resulting in a time-varying level-1 (within-person) predictor and a time-invariant level-2 (between-person) predictor that were included together in the model.

The initial model included fixed effects for age group, the time-invariant between-person sleep predictor, the time-varying within-person sleep predictor and the covariates, as well as a random intercept. Models were initially estimated using maximum likelihood to allow likelihood ratio testing. We then determined the best-fitting random effects and residual structure by testing whether adding an autoregressive AR1 residual structure and a random slope for the within-person time-varying sleep predictor significantly (*p* < .05) improved model fit, based on likelihood ratio tests. The best-fitting model served as baseline model. Results from fitting this baseline model are presented in Supplementary Material S1).

We then tested the hypothesis that age group moderated the effect of the sleep predictor on cognitive performance in terms of processing speed. Two models were fitted that were compared against the baseline model using maximum likelihood testing with a significance level of *p* < .05. Interaction model 1 extended the baseline model by including both (1) the interaction of age group and the within-person effect of the sleep predictor, and (2) the interaction of age group and the between-person effect of the sleep predictor. Interaction model 2 included only the age group and within-person effect of the sleep predictor as extension of the baseline model.

For sleep duration, we additionally tested whether adding quadratic terms for both the time-invariant and time-varying predictor for sleep duration significantly improved the model fit. This step accounted for the possibility of a nonlinear relationship between sleep duration and processing speed, as previous research suggests that both very short and very long sleep duration may be associated with worse performance [15, 39].

The best-fitting model for each sleep-predictor was re-fitted with restricted maximum likelihood to obtain unbiased parameter estimates.

## Results

### Descriptive statistics for the main study variables

Table 2 shows the descriptive statistics for the main study variables across the two age groups. On average, in the young adults group, 19.66 days with actigraphy and 19.59 days with sleep diaries were included in the analysis. In the older adult group, on average 20.58 days with actigraphy and 20.48 days with sleep diaries were included. The number of daily observations varied between 6406 and 6474 across analyses due to missing data.

**Table 2:** Descriptive results for main study variables across the age groups (mean (standard deviation))

	**Young adults**	**Older adults**	**Overall**
Completed actigraphy days^a^	19.66 (2.32)	20.58 (1.36)	
Completed sleep diary days^b^	19.59 (2.35)	20.48 (1.25)	
Sleep duration mean (h)^a^	6.27 (0.60)	6.23 (0.71)	
Sleep duration iSD (h)^a^	1.01 (0.29)	0.79 (0.26)	
Sleep duration ICC^a^			.306
Sleep efficiency mean (%)^a^	78.77 (5.41)	79.56 (6.88)	
Sleep efficiency iSD (%)^a^	5.12 (2.13)	4.65 (1.93)	
Sleep efficiency ICC^a^			.575
Sleep quality index mean^b^	1.85 (0.42)	1.96 (0.46)	
Sleep quality index iSD^b^	0.58 (0.20)	0.63 (0.22)	
Sleep quality index ICC^b^			.299
Number of correct responses mDSST mean^c^	30.87 (5.37)	26.54 (3.35)	
Number of correct responses mDSST iSD^c^	2.30 (0.92)	1.76 (0.44)	
mDSST ICC^c^			.840
Average percentage correct responses mDSST^c^	97.77 (2.45)	98.28 (1.26)	
Average number of mDSST completed per day^c^	5.04 (1.13)	5.95 (1.14)	

iSD = intra-individual standard deviation, ICC = intraclass correlation coefficient, representing the proportion of between-subject variance relative to total variance, based on a null model with random intercept only, mDSST = momentary Digit Symbol Substitution Task.

a includes data from participants who completed at least 10 nights with actigraphy.

b includes data from participants who completed at least 10 nights with sleep diary.

c includes data from participants who completed at least 10 nights with actigraphy and/or 10 nights with sleep diary.

Young participants had an average sleep duration of 6.27 h and older participants of 6.23 h. As indicated by the intra-individual standard deviations of 1.01 h in the young adults and 0.79 h in the older adults, sleep duration varied on average considerably within-person in both age groups.

Young adults had a mean subjective sleep quality score of 1.85, and older adults had a mean score of 1.96, on a scale from 1 to 5, where lower scores indicate better sleep quality. The average number of correct responses per day in the mDSST was 30.87 items for the young adults and 26.54 items for the older adults, with both groups having high accuracy.

### Sleep duration predicting next-day processing speed

For sleep duration, the baseline model without interactions between age group and the between-person and within-person component of sleep duration provided the best fit (see Table 3).

**Table 3:** Results from model-fitting testing the interactions between age group and the sleep predictors. Selected models are marked in bold

	**Log-likelihood**	**df**	**AIC**	***p* comparison against baseline model**
**Sleep duration**				
**Baseline model**	−13 863.47	11	27 748.94	
Interaction model 1	−13 862.42	13	27 750.84	.350
Interaction model 2	−13 863.47	12	27 750.94	.974
Quadratic model	−13 862.41	13	27 750.81	.345
**Sleep efficiency**				
**Baseline model**	−13 838.44	11	27 698.87	
Interaction model 1	−13 837.08	13	27 700.17	.259
Interaction model 2	−13 837.44	12	27 698.88	.158
**Sleep quality**				
**Baseline model**	−14 061.69	11	28 145.39	
Interaction model 1	−14 061.23	13	28 148.46	.628
Interaction model 2	−14 061.45	12	28 146.9	.485

df = degrees of freedom; AIC = Akaike information criterion, *p = p-*value

Baseline models included age group, the between-person and within-person component of the respective sleep predictor, a random intercept and an autoregressive (AR1) residual structure. Interaction Model 1 included in addition interaction terms between age group and both the between and within-person component of the sleep predictor. Interaction Model 2 included in addition to the baseline model the interaction term between age group and the within-person component of the sleep predictor. The quadratic model for sleep duration included in addition to the baseline model quadratic terms for the between and within-person components of sleep duration as fixed effects. All models were adjusted for the covariates sex, full-time retirement, study day, and study day^2.

At the within-person level, sleeping less than one’s own mean sleep duration was significantly associated with poorer performance, while sleeping longer was associated with better performance (see Table 4). Including quadratic terms for sleep duration did not improve the model fit (see Table 3), suggesting that this effect of sleep duration on performance was linear. At the between-person level, there was no significant association between mean sleep duration with performance on the mDSST. Older adults had significantly fewer numbers of correct responses.

**Table 4:** Multilevel model with sleep duration and age group predicting next-day average number of correct responses in the mDSST (*n* = 6423 observations with 319 participants and an average of 20.1 observations per participant)

	**Estimate (95% confidence interval)**
Older adult group vs young adult group	−3.51^***^
	[−4.59 −2.43]
Between person effect: mean sleep duration (h)	0.30
	[−0.44 1.05]
Within-person effect: sleep duration deviation (h)	0.11^***^
	[0.06 0.15]

Results are presented as unstandardized regression coefficients. ^*^p < .05. ^**^p < .01. ^***^p < .001. The model was adjusted for the covariates sex, full-time retirement, study day, study day^2, and included a random intercept and an autoregressive residual (AR1) structure.

**Figure 2:**
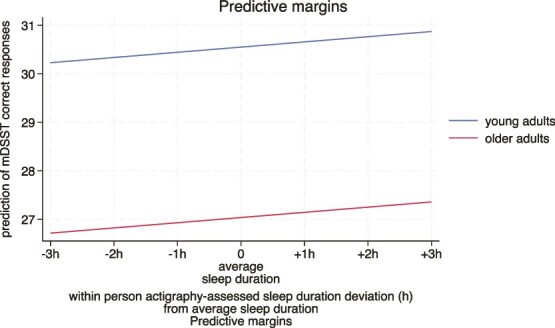
Predictive marginal means showing the within-person association between deviations from a person’s own average sleep duration and the mean number of correct responses in the momentary Digit Symbol Substitution Task (mDSST) on the next day. Note: 0 on the *x*-axis represents each person’s average sleep duration, and values reflect deviations from that individual baseline (i.e. more or less sleep than usual).

Figure 2 displays the marginal means for the number of correct responses in the mDSST by within-person sleep duration deviation in young and older adults.

Full model results, including covariates and random effects, are presented in Supplementary Material S2a.

### The effect of sleep efficiency on next-day processing speed

The baseline model without interactions between age group and the between-person and within-person component of sleep efficiency provided the best fit (see Table 3).

Neither within-person nor between-person differences in sleep efficiency were significantly associated with the number of correct responses in the mDSST (see Table 5). Older adults had on average significantly fewer correct responses in the mDSST. Full model results including covariates are presented in Supplementary Material S2b.

**Table 5:** Multilevel model with sleep efficiency and age group predicting next-day average number of correct responses in the mDSST (*n* = 6406 observations with 319 participants and an average of 20.1 observations per participant)

	**Estimate (95% confidence interval)**
Older adult group vs young adult group	−3.57^***^
	[−4.65 −2.49]
Between person effect: mean sleep efficiency (%)	0.03
	[−0.05 0.11]
Within-person effect: sleep efficiency deviation (%)	0.00
	[−0.01 0.01]

Results are presented as unstandardized regression coefficients. ^*^p < .05. ^**^p < .01. ^***^p < .001. The model was adjusted for the covariates sex, full-time retirement, study day, study day^2, and included a random intercept and an autoregressive residual (AR1) structure. Sleep efficiency was not included for nights with split night sleep and, due to technical issues, nights including spring time daylight saving time transitions.

### Sleep quality predicting next-day processing speed

For sleep quality, the baseline model without interactions between age group and sleep quality provided the best fit (see Table 3).

At the within-person level, subjective sleep quality was significantly associated with next-day performance in the mDSST. A 1-unit poorer sleep quality rating relative to a person’s own mean level was associated with on average 0.08 fewer correct responses the following day (see Table 6). In contrast, at the between-person level, mean sleep quality across the 21 days was not significantly associated with the number of correct responses on the mDSST. Older adults showed significantly worse performance compared to younger adults. Full model results including covariates are presented in Supplementary Material S2c.

**Table 6:** Multilevel model with sleep quality and age group predicting next-day average number of correct responses in the mDSST (*n* = 6474 observations with 323 participants and an average of 20.0 observations per participant)

	**Estimate (95% confidence interval)**
Older adult group vs. young adult group	−3.40^***^
	[−4.51 −2.30]
Between person effect: mean sleep quality	−0.60
	[−1.67 0.48]
Within-person effect: sleep quality deviation	−0.08^*^
	[−0.15 −0.01]

Results are presented as unstandardized regression coefficients. ^*^p < .05. ^**^p < .01. ^***^p < .001. The model was adjusted for the covariates sex, full-time retirement, study day, study day^2, and included a random intercept and an autoregressive residual (AR1) structure.

## Discussion

This study investigated the effect of actigraphically measured sleep duration, sleep efficiency, and subjective sleep quality on next-day performance in a processing speed task among young and older adults over a 21-day period in a real-life setting. The results showed that shorter than usual sleep duration and poorer than usual subjective sleep quality—but not reduced sleep efficiency—were associated with significantly worse performance the following day. In contrast, between-person differences in sleep duration, sleep efficiency, and sleep quality were not significantly related to processing speed. While older adults generally had poorer processing speed, contrary to the hypotheses, they were not significantly less affected by daily variations in sleep.

### Within-person effects of sleep duration, sleep efficiency, and sleep quality on processing speed

Sleeping shorter or longer than one’s mean sleep duration on a given night was associated with slightly poorer or better performance in the mDSST the following day, respectively, confirming our hypothesis. While this finding aligns well with experimental studies showing that both total [11] and partial sleep deprivation [13] impair processing speed assessed on the DSST, the estimated effect of approximately 0.1 difference in correct responses per hour of sleep difference was smaller than the 0.8 difference that we expected a priori [28], which was based on a previous laboratory study of partial sleep deprivation [13]. The smaller impact of sleep duration observed in this real-world study, compared to what we expected based on previous laboratory findings [13], may at least in part be due to contextual factors such as company, sounds and other distractions [40] that affect performance in real-world conditions. While these contextual factors likely introduced variability in the performance measures, the present results enhance our understanding of the impact of day-to-day variations in sleep within the complexities and noise of natural daily life, and thus provide insights on the real-world relevance [23]. Our results are in line with a recent study in adolescents, which found that next-day performance in the PVT deteriorated with shorter sleep duration the prior night, albeit with a very small effect size [25]. Likewise, in children, time in bed correlated with next-day working memory performance in the morning [24]. Moreover, a study in older adults found an association between daily sleep duration and working memory performance, although only in individuals who slept relatively little on average [26].

In contrast to our hypothesis, the results did not show any within-person effect of sleep efficiency on next-day processing speed. This is also in contrast to findings in adolescents, where daily higher sleep efficiency predicted faster reaction times in the Psychomotor Vigilance Task, albeit with a very small effect size [25]. The reasons for these divergent results are unclear but may relate to differences in the cognitive tasks used or the age groups studied.

Notably, some studies have found that longer wake times at night are paradoxically associated with better cognitive performance in older adults [41]. When interpreting the lack of effect in the current study, it is important to also consider the complexity of sleep efficiency as a measure. Sleep efficiency, unlike sleep duration and sleep quality, can be a more ambiguous indicator. While high sleep efficiency is usually considered an indicator of good sleep, high sleep efficiency could also be due to short sleep duration and high sleep pressure, rather than genuinely reflecting better sleep on a given night.

In line with our hypothesis, having poorer sleep quality than usual on a given night was associated with poorer performance on the next day. This matches the results obtained in children, where working memory performance was associated with self-reported sleep quality the previous night [24].

### Between-person effects of sleep duration, sleep efficiency, and sleep quality on processing speed

Although the main aim of this study was to examine the within-person effects of the sleep variables on next-day cognitive performance in terms of processing speed, and to test the cross-level interaction between age group and the within-person effects, between-person effects of the sleep predictors were included in the analyses. In contrast to the observed within-person effects of sleep duration and sleep quality on next-day processing speed, the results did not show any significant between-person effects of sleep duration, sleep efficiency, or sleep quality. This suggests that individuals who typically sleep shorter or longer, or more poorly or better than others did not, on average, differ in their performance in the mDSST. This underscores the importance of distinguishing within-person and between-person effects of sleep. The lack of between-person effects suggests that variation in average sleep duration across individuals is not associated with processing speed. This might be due to that individuals, on average, achieve a sufficient level of sleep. Yet this interpretation is challenged by the within-person effects, which indicate that sleeping somewhat longer than usual still positively impacts next-day processing speed.

Instead, this may suggest that it is the default for many individuals, on average, to sleep less than their optimal sleep duration. The lack of between-person association in our study is in accordance with meta-analyses that, based on mostly cross-sectional studies, found no association between self-reported sleep duration [42] and objective sleep measures [16] with processing speed in older adults. In contrast, Lucke, Wrzus, Gerstorf, Kunzmann, Katzorreck, Schmiedek, Hoppmann, and Schilling [26] reported a between-person association between self-reported sleep duration and working memory performance in their daily life study in older adults. The discrepancy between the findings of Lucke et al.’s study and the present results may be due to several factors: Lucke et al.’s study relied on self-reported sleep measures, which do not align well with objective sleep indicators [43, 44]. Moreover, the studies examined different cognitive domains which may vary in their sensitivity to sleep variations. Specifically, self-reported sleep duration has been associated with working memory, but not with processing speed, according to a meta-analysis in older adults [42]. Finally, the age ranges differed between studies: Lucke et al’s [26] sample included adults aged 66–90 years while the present study included both young adults aged 18–30 years and adults aged 55–75 years.

The lack of between-person effects of sleep quality on cognitive performance in the present study aligns well with previous findings showing that poor habitual sleep quality is not linked with processing speed in older adults [45, 46]. Similarly, Zavecz, Nagy, Galko, Nemeth, and Janacsek [47] found no association between habitual sleep quality and the cognitive functions they assessed, including working memory, executive functions, and procedural learning, in young adults.

### Impact of age group

In line with a wealth of previous studies using the DSST [48, 49], we found that performance in the mDSST was significantly worse in older adults compared with young adults. This finding underscores the utility of remote cognitive testing in real-world contexts for investigating conditions or groups that exhibit more pronounced changes in cognitive ability. Yet, contrary to our hypotheses, the effect of daily variations in sleep duration, sleep efficiency, and sleep quality on next-day processing speed did not significantly differ between young and older adults. This is in discordance with previous laboratory-based studies, which showed that young adults are more affected by sleep deprivation than older adults in sustained attention tasks [4, 5]. Several factors, beyond the difference between the laboratory and real-world environment, may contribute to the absence of age-moderation effects in this study: On the one hand, the age differences in response to sleep loss could be domain specific. For instance, Sagaspe, Taillard, Amieva, Beck, Rascol, Dartigues, Capelli, and Philip [21] found that extended wakefulness affected simple attention but not inhibitory control differently in young and older males. While the mDSST is less complex than tasks measuring inhibitory control, it is more complex than simple sustained attention tasks. As such, differences in task demands may contribute to the heterogenous findings across studies. On the other hand, it may be that the strict selection criteria often applied in laboratory studies result in a selection bias particularly in the older age group. While our study still required participants to be free of health conditions and/or medical treatments likely to significantly affect daily physical activity, sleep, psychological well-being, or performance, we did not require them to be entirely medication-free or free of all chronic health conditions, as previous sleep deprivation studies have done [4, 21]. The very strict selection criteria used in previous studies may be less of an issue in younger samples, where participants are probably more likely to meet the health requirements. Yet in older adults, these criteria likely resulted in a highly selective group, which may partly explain their lower observed vulnerability in the sleep deprivation studies.

### Strengths and limitations

This study, the first to examine the impact of day-to-day variations in sleep on cognitive performance in both young and older adults within a real-world context, has several strengths. The large sample size, combined with the 21-day study period, results in a large dataset with more than 6000 observations. A strength of the study is also the use of the DSST, a well-established cognitive task that is also known to be sensitive to experimental sleep loss [11, 13, 14]. In addition, the app-based version of this task has been successfully used in naturalistic environments before [31, 40]. A strength is also the use of actigraphy as an objective measure of sleep duration and efficiency, alongside subjective assessments of sleep quality.

Yet, there are also several limitations to consider. While actigraphy is a valid and reliable method for objectively measuring sleep [50], it does not capture sleep architecture and microstructure, which are known to vary more with age than sleep duration [19, 20], and have been linked to cognitive outcomes. For example, slow-wave sleep has been associated positively with processing speed [51], and in older adults, polysomnography-derived metrics related to rapid eye movement duration, electroencephalographic power spectra, sleep spindles, and slow oscillations have been shown to be associated to processing speed independently of changes in sleep quality or quantity [52].

Moreover, when interpreting the current results it needs to be considered that the effect of sleep duration on performance was smaller than we expected in the a priori power analysis [28]. This may imply that despite the large sample size the statistical power may have been not sufficient to detect more subtle effects for the interaction between age group and daily sleep variations, while statistical power for within-person main effects very likely was still high. The study may also not have had sufficient power to detect subtle between-person effects of sleep duration, efficiency, and quality. When interpreting the results, it is important to consider, that although we used less restrictive inclusion criteria than experimental sleep deprivation studies, individuals with severe health conditions were still excluded. Another limitation of the study is the relatively young age range of the older adult group (55–75 years), which may have limited our ability to detect more pronounced age effects. Future studies should therefore consider including even older age groups than those examined in the present study.

Moreover, the present study focuses on the impact of variations in the previous night’s sleep on processing speed, without considering cumulative effects of consecutive nights of shorter sleep, which have been previously shown for processing speed in the laboratory [13]. In the current analysis, we used an aggregated, average performance measure across the day, which we consider a meaningful indicator of overall processing speed. Nevertheless, there may be time-of-day–specific associations between daily variations in sleep, processing speed, and adult age that are not captured by the current analytic approach. A further limitation of the study is that participants may have compensated for short or poor sleep by allocating additional cognitive resources to the task, particularly given the short task duration. It is also possible that effects on processing speed may only become evident when sleep is reduced by several hours. Yet such pronounced reductions, typically required in experimental sleep deprivation protocols, are rare in field studies, as most participants likely try to obtain adequate sleep duration.

Future studies should investigate whether longer tasks might better capture subtle impairments in processing speed associated with day-to-day sleep variations. Finally, results may not be generalizable to other cognitive domains beyond processing speed, as the effects of both total [53] and partial sleep deprivation [2] are known to vary across cognitive domains.

## Conclusion

Using an intensive longitudinal design with daily measures of sleep and processing speed across 21 days in more than 300 individuals, this study shows that day-to-day fluctuations in sleep duration and subjective sleep quality have a significant impact on next-day processing speed when assessed in real-world contexts. Of note, the observed effects on cognitive performance were smaller than expected, suggesting that day-to-day changes in sleep may have a more limited impact [28] on brief cognitive tests in the presence of contextual factors inherent to real-life conditions. Consistent with prior research, processing speed was significantly worse in older adults compared to young adults, indicating that remote mobile testing in everyday life could be a viable method for identifying cognitive differences across age groups. Contrary to our hypothesis, the relationship between sleep and performance in the processing speed task did not differ significantly between young and older adults. This highlights the importance of maintaining adequate sleep each day for cognitive functioning in real-world situations, regardless of age.

## Supplementary Material

Supplementary_zsaf321

## Data Availability

The data underlying this article cannot be shared publicly due to GDPR restrictions. The data and analysis code will be shared upon reasonable request to the corresponding author.

## References

[1] Pilcher JJ, Huffcutt AI. Effects of sleep deprivation on performance: a meta-analysis. *Sleep*. 1996;19(4):318–326. 10.1093/sleep/19.4.318 8776790

[2] Lowe CJ, Safati A, Hall PA. The neurocognitive consequences of sleep restriction: a meta-analytic review. *Neurosci Biobehav Rev*. 2017; 80:586–604. 10.1016/j.neubiorev.2017.07.010 28757454

[3] Lim J, Tan JC, Parimal S, Dinges DF, Chee MW. Sleep deprivation impairs object-selective attention: a view from the ventral visual cortex. *PLoS One*. 2010;5(2):ef9087. 10.1371/journal.pone.0009087 PMC2816724 20140099

[4] Duffy JF, Willson HJ, Wang W, Czeisler CA. Healthy older adults better tolerate sleep deprivation than young adults. *J Am Geriatr Soc*. 2009;57(7):1245–1251. 10.1111/j.1532-5415.2009.02303.x 19460089 PMC3122254

[5] Philip P, Taillard J, Sagaspe P, et al. Age, performance and sleep deprivation. *J Sleep Res*. 2004;13(2):105–110. 10.1111/j.1365-2869.2004.00399.x 15175089

[6] Messman BA, Wiley JF, Yap Y, et al. How much does sleep vary from night-to-night? A quantitative summary of intraindividual variability in sleep by age, gender, and racial/ethnic identity across eight-pooled datasets. *J Sleep Res*. 2022;31(6):e13680. 10.1111/jsr.13680 35811092 PMC9649840

[7] Bei B, Wiley JF, Trinder J, Manber R. Beyond the mean: a systematic review on the correlates of daily intraindividual variability of sleep/wake patterns. *Sleep Med Rev*. 2016;28:108–124. 10.1016/j.smrv.2015.06.003 26588182

[8] Saklofske DH, Breaux K, Beal AL, Raiford SE, Weiss LG. Chapter 9 - WISC–V and the Evolving Role of Intelligence Testing in the Assessment of Learning Disabilities. In: Weiss LG, Saklofske DH, Holdnack JA, Prifitera A, eds. WISC-V (Second Edition). Academic Press; 2019:271–336. 10.1016/B978-0-12-815744-2.00009-4

[9] Jaeger J . Digit Symbol Substitution Test: the case for sensitivity over specificity in neuropsychological testing. *J Clin Psychopharmacol*. 2018;38(5):513–519. 10.1097/JCP.0000000000000941 30124583 PMC6291255

[10] Salthouse TA . The processing-speed theory of adult age differences in cognition. *Psychol Rev*. 1996;103(3):403–428. 10.1037/0033-295x.103.3.403 8759042

[11] Honn KA, Halverson T, Jackson ML, et al. New insights into the cognitive effects of sleep deprivation by decomposition of a cognitive throughput task. *Sleep*. 2020;43(7). 10.1093/sleep/zsz319 PMC7355397 32227081

[12] Yamazaki EM, Antler CA, Lasek CR, Goel N. Residual, differential neurobehavioral deficits linger after multiple recovery nights following chronic sleep restriction or acute total sleep deprivation. *Sleep*. 2021;44(4). 10.1093/sleep/zsaa224 PMC8274462 33274389

[13] Van Dongen HP, Maislin G, Mullington JM, Dinges DF. The cumulative cost of additional wakefulness: dose-response effects on neurobehavioral functions and sleep physiology from chronic sleep restriction and total sleep deprivation. *Sleep*. 2003;26(2):117–126. 10.1093/sleep/26.2.117 12683469

[14] Banks S, Van Dongen HP, Maislin G, Dinges DF. Neurobehavioral dynamics following chronic sleep restriction: dose-response effects of one night for recovery. *Sleep*. 2010;33(8):1013–1026. 10.1093/sleep/33.8.1013 20815182 PMC2910531

[15] Richards A, Inslicht SS, Metzler TJ, et al. Sleep and cognitive performance from teens to old age: more is not better. *Sleep*. 2017;40(1). 10.1093/sleep/zsw029 PMC6251526 28364476

[16] Qin S, Leong RLF, Ong JL, Chee MWL. Associations between objectively measured sleep parameters and cognition in healthy older adults: a meta-analysis. *Sleep Med Rev*. 2023;67:101734. 10.1016/j.smrv.2022.101734 36577339

[17] Sakal C, Li T, Li J, Yang C, Li X. Association between sleep efficiency variability and cognition among older adults: cross-sectional accelerometer study. *JMIR Aging*. 2024;7:e54353. 10.2196/54353 38596863 PMC11007383

[18] Zhang B, Wing YK. Sex differences in insomnia: a meta-analysis. *Sleep*. 2006;29(1):85–93. 10.1093/sleep/29.1.85 16453985

[19] Ohayon MM, Carskadon MA, Guilleminault C, Vitiello MV. Meta-analysis of quantitative sleep parameters from childhood to old age in healthy individuals: developing normative sleep values across the human lifespan. *Sleep*. 2004;27(7):1255–1273. 10.1093/sleep/27.7.1255 15586779

[20] Schwarz JF, Akerstedt T, Lindberg E, Gruber G, Fischer H, Theorell-Haglow J. Age affects sleep microstructure more than sleep macrostructure. *J Sleep Res*. 2017;26(3):277–287. 10.1111/jsr.12478 28093830

[21] Sagaspe P, Taillard J, Amieva H, et al. Influence of age, circadian and homeostatic processes on inhibitory motor control: a Go/Nogo task study. *PLoS One*. 2012;7(6):e39410. 10.1371/journal.pone.0039410 22761784 PMC3382614

[22] Cai AWT, Manousakis JE, Singh B, et al. On-road driving impairment following sleep deprivation differs according to age. *Sci Rep*. 2021;11(1):21561. 10.1038/s41598-021-99133-y 34732793 PMC8566466

[23] Moore RC, Swendsen J, Depp CA. Applications for self-administered mobile cognitive assessments in clinical research: a systematic review. *Int J Methods Psychiatr Res*. 2017;26(4):1562. 10.1002/mpr.1562 PMC5623609 28370881

[24] Konen T, Dirk J, Schmiedek F. Cognitive benefits of last night's sleep: daily variations in children's sleep behavior are related to working memory fluctuations. *J Child Psychol Psychiatry*. 2015;56(2):171–182. 10.1111/jcpp.12296 25052368

[25] Shen L, Nicolazzo J, Sletten TL, et al. Daily fluctuations in adolescents' sleep predict next-day attention, sleepiness, and fatigue: an ecological momentary assessment study over 28 days. *J Child Psychol Psychiatry*. 2024;66(5):686–696. 10.1111/jcpp.14076 39618031 PMC12018295

[26] Lucke AJ, Wrzus C, Gerstorf D, et al. Between-person and within-person associations of sleep and working-memory in the everyday lives of old and very old adults: initial level, learning, and variability. *Sleep*. 2022;45(1). 10.1093/sleep/zsab279 34922403

[27] Schwarz J, Gerhardsson A, Kecklund G, et al. Mood impairment is less strong in older than in young adults after sleep deprivation. *J Sleep Res*. 2018;28(4):e12801. 10.1111/jsr.12801 30585371 PMC7379256

[28] Schwarz J, Freidle M, van Leeuwen W, Åkerstedt T, Kecklund G. Sleep in everyday life—relationship to mood and performance in young and older adults: a study protocol. *Front Psychol*. 2023;14:1264881. 10.3389/fpsyg.2023.1264881 38078262 PMC10701737

[29] Elbaz M, Yauy K, Metlaine A, Martoni M, Leger D. Validation of a new actigraph motion watch versus polysomnography on 70 healthy and suspected sleep disordered subjects. *J Sleep Res*. 2012;21:218

[30] Åkerstedt T, Hume K, Minors D, Waterhouse J. Good sleep—its timing and physiological sleep characteristics. *J Sleep Res*. 1997; 6(4):221–229. 10.1111/j.1365-2869.1997.00221.x 9493521

[31] Verhagen SJW, Daniels NEM, Bartels SL, et al. Measuring within-day cognitive performance using the experience sampling method: a pilot study in a healthy population. *PLoS One*. 2019;14(12):e0226409. 10.1371/journal.pone.0226409 31830099 PMC6907820

[32] Roivainen E . Gender differences in processing speed: a review of recent research. *Learn Individ Differ*. 2011;21(2):145–149. 10.1016/j.lindif.2010.11.021

[33] Bixler EO, Papaliaga MN, Vgontzas AN, et al. Women sleep objectively better than men and the sleep of young women is more resilient to external stressors: effects of age and menopause. *J Sleep Res*. 2009;18(2):221–228. 10.1111/j.1365-2869.2008.00713.x 19302341 PMC3594776

[34] Gosselin C, Boller B. The impact of retirement on executive functions and processing speed: findings from the Canadian Longitudinal Study on Aging. *Neuropsychol Dev Cogn B Aging Neuropsychol Cogn*. 2024;31(1):1–15. 10.1080/13825585.2022.2110562 35996815

[35] Myllyntausta S, Stenholm S. Sleep before and after retirement. *Curr Sleep Med Rep*. 2018;4(4):278–283. 10.1007/s40675-018-0132-5 30464885 PMC6223890

[36] Benyamini Y . Why does self-rated health predict mortality? An update on current knowledge and a research agenda for psychologists. *Psychol Health*. 2011;26(11):1407–1413. 10.1080/08870446.2011.621703 22111660

[37] Hoffman L, Stawski RS. Persons as contexts: evaluating between-person and within-person effects in longitudinal analysis. *Res Hum Dev*. 2009;6(2–3):97–120. 10.1080/15427600902911189

[38] Wang LP, Maxwell SE. On disaggregating between-person and within-person effects with longitudinal data using multilevel models. *Psychol Methods*. 2015;20(1):63–83. 10.1037/met0000030 25822206

[39] Kronholm E, Sallinen M, Suutama T, Sulkava R, Era P, Partonen T. Self-reported sleep duration and cognitive functioning in the general population. *J Sleep Res*. 2009;18(4):436–446. 10.1111/j.1365-2869.2009.00765.x 19732318

[40] Daniels NEM, Bartels SL, Verhagen SJW, Van Knippenberg RJM, De Vugt ME, Delespaul P. Digital assessment of working memory and processing speed in everyday life: feasibility, validation, and lessons-learned. *Internet Interv*. 2020;19:100300. 10.1016/j.invent.2019.100300 31970080 PMC6965714

[41] Scullin MK, Bliwise DL. Sleep, cognition, and normal aging: integrating a half-century of multidisciplinary research. *Perspect Psychol Sci*. 2015;10(1):97–137. 10.1177/1745691614556680 25620997 PMC4302758

[42] Lo JC, Groeger JA, Cheng GH, Dijk DJ, Chee MW. Self-reported sleep duration and cognitive performance in older adults: a systematic review and meta-analysis. *Sleep Med*. 2016;17:87–98. 10.1016/j.sleep.2015.08.021 26847980

[43] Lehrer HM, Yao Z, Krafty RT, et al. Comparing polysomnography, actigraphy, and sleep diary in the home environment: the study of Women’s Health Across the Nation (SWAN) Sleep Study. *Sleep Adv*. 2022;3(1):zpac001. 10.1093/sleepadvances/zpac001 35296109 PMC8918428

[44] Matthews KA, Patel SR, Pantesco EJ, et al. Similarities and differences in estimates of sleep duration by polysomnography, actigraphy, diary, and self-reported habitual sleep in a community sample. *Sleep Health*. 2018;4(1):96–103. 10.1016/j.sleh.2017.10.011 29332687 PMC5771411

[45] Miyata S, Noda A, Iwamoto K, Kawano N, Okuda M, Ozaki N. Poor sleep quality impairs cognitive performance in older adults. *J Sleep Res*. 2013;22(5):535–541. 10.1111/jsr.12054 23560612

[46] Nebes RD, Buysse DJ, Halligan EM, Houck PR, Monk TH. Self-reported sleep quality predicts poor cognitive performance in healthy older adults. *J Gerontol B Psychol Sci Soc Sci*. 2009;64B(2):180–187. 10.1093/geronb/gbn037 PMC2655169 19204069

[47] Zavecz Z, Nagy T, Galko A, Nemeth D, Janacsek K. The relationship between subjective sleep quality and cognitive performance in healthy young adults: evidence from three empirical studies. *Sci Rep*. 2020;10(1):4855. 10.1038/s41598-020-61627-6 32184462 PMC7078271

[48] Hoyer WJ, Stawski RS, Wasylyshyn C, Verhaeghen P. Adult age and digit symbol substitution performance: a meta-analysis. *Psychol Aging*. 2004;19(1):211–214. 10.1037/0882-7974.19.1.211 15065945

[49] Salthouse TA . What do adult age differences in the Digit Symbol Substitution Test reflect? *J Gerontol*. 1992;47(3):P121–P128. 10.1093/geronj/47.3.P121 1573192

[50] Sadeh A . The role and validity of actigraphy in sleep medicine: an update. *Sleep Med Rev*. 2011;15(4):259–267. 10.1016/j.smrv.2010.10.001 21237680

[51] Della Monica C, Johnsen S, Atzori G, Groeger JA, Dijk DJ. Rapid eye movement sleep, sleep continuity and slow wave sleep as predictors of cognition, mood, and subjective sleep quality in healthy men and women, aged 20-84 years. *Front Psych*. 2018;9:255. 10.3389/fpsyt.2018.00255 PMC6024010 29988413

[52] Djonlagic I, Mariani S, Fitzpatrick AL, et al. Macro and micro sleep architecture and cognitive performance in older adults. *Nat Hum Behav*. 2021;5(1):123–145. 10.1038/s41562-020-00964-y 33199858 PMC9881675

[53] Lim J, Dinges DF. A meta-analysis of the impact of short-term sleep deprivation on cognitive variables. *Psychol Bull*. 2010;136(3):375–389. 10.1037/a0018883 20438143 PMC3290659

