# Supplementary material for: Daily fluctuations in sleep duration and quality affect next-day processing speed performance in young and older adults: an intensive longitudinal everyday life study over 21 days
Source: Sleep. 2025 Oct 14;49(1):zsaf321. doi: 10.1093/sleep/zsaf321 (PMC12795734; doi:10.1093/sleep/zsaf321)
Supplement: Supplementary_zsaf321 [file supplementary_zsaf321.pdf]

Supplementary Material

Daily fluctuations in sleep duration and quality affect next-day processing speed performance in young and older adults: an intensive longitudinal everyday life study over 21 days

Johanna Schwarz[1,2], Malin Freidle[1], Wessel van Leeuwen[1], Jade Silfverling[1], Torbjörn Åkerstedt[1,2] & Göran Kecklund[1]

[1] Department of Psychology, Stockholm University, Stockholm, Sweden

[2] Department of Clinical Neuroscience, Karolinska Institute, Stockholm, Sweden

Corresponding Author:

Johanna Schwarz

Department of Psychology

Stockholm University

106 91 Stockholm

Sweden

johanna.schwarz@su.se

S1: Results from model fitting step 1: determining the residual and random effects structure

Supplementary Table S1. Results from model fitting step 1

	Log-likelihood	df	AIC	*P* Comparison against M1	P Comparison against M2
Sleep duration					
M1a Initial Model	-14067.14	10	28154.27		
**M1b Initial Model + autoregressive (AR1) residual structure**	**-13863.47**	**11**	**27748.94**	**< .001**	
M1c Initial Model + + autoregressive (AR1) residual structure + Random slope for sleep duration deviation	-13862.61	12	27749.23		0.191
Sleep efficiency					
M1a Initial Model	-14037.46	10	28094.92		
**M1b Initial Model + autoregressive (AR1) residual structure**	**-13838.44**	**11**	**27698.87**	**< .001**	
M1c Initial Model + autoregressive (AR1) residual structure + Random slope for sleep duration deviation	-13838.44	12	27700.87		1
Sleep quality					
M1a Initial Model	-14298.65	10	28617.31		
**M1b Initial Model + autoregressive (AR1) residual structure**	**-14061.69**	**11**	**28145.39**	**< .001**	
M1c Initial Model + +autoregressive (AR1) residual structure+ Random slope for sleep duration deviation	Not converged				

Bold print depicts the baseline model used in step 2 of the model fitting

S2a-c: Full model specifications including covariates (main analysis)

Table S2a: Multilevel mixed modelling examining the effect of prior night sleep duration (actigraphy-based) as predictor of next day average number of correct responses in the mDSST

Older adult group vs young adult group	-3.51	***
	[-4.59 -2.43]	
Average sleep duration (h) (between person effect)	0.30	
	[-0.44 1.05]	
Sleep duration (h) deviation (within person effect)	0.11	***
	[0.06 0.15]	
Study day	0.35	***
	[0.31 0.39]	
Study day quadratic	-0.01	***
	[-0.01 -0.01]	
Male	-1.63	**
	[-2.62 -0.65]	
Fulltime retired	-2.43	***
	[-3.74 -1.12]	
Intercept	27.66	***
	[22.77 32.55]	
var(_cons)	17.39	
	[14.83 20.40]	
var(e)	3.91	***
	[3.75 4.07]	
rho	0.29	***
	[0.26 0.32]	
Number of observations	6423	

Results are presented as unstandardised regression coefficients and 95% confidence intervals. * p <.05 ** p < .01 *** p < .001

Table S2b: Multilevel mixed modelling examining the effect of prior night sleep efficiency (actigraphy-based) as predictor of next day average number of correct responses in the mDSST

Older adult group vs young adult group	-3.57	***
	[-4.65 -2.49]	
Average sleep efficiency (%) (between person effect)	0.03	
	[-0.05 0.11]	
Sleep efficiency (%) deviation (within person effect)	0.00	
	[-0.01 0.01]	
Study day	0.35	***
	[0.30 0.39]	
Study day quadratic	-0.01	***
	[-0.01 -0.01]	
Male	-1.67	***
	[-2.64 -0.71]	
Fulltime retired	-2.38	***
	[-3.69 -1.07]	
Intercept	27.35	***
	[21.11 33.59]	
var(_cons)	17.42	
	[14.85 20.43]	
var(e)	3.92	***
	[3.76 4.08]	
rho	0.29	***
	[0.26 0.31]	
Number of observations	6406	

Results are presented as unstandardised regression coefficients and 95% confidence intervals. * p <.05 ** p < .01 *** p < .001

Table S2c: Multilevel mixed modelling examining the effect of prior night sleep quality as predictor of next day average number of correct responses in the mDSST

Older adult group vs young adult group	-3.40<br>[-4.51 -2.30]	***
Average sleep quality index (between person effect)	-0.60<br>[-1.67 0.48]	
Sleep quality index deviation (within person effect)	-0.08<br>[-0.15 -0.01]	*
Study day	0.35<br>[0.30 0.39]	***
Study day quadratic	-0.01<br>[-0.01 -0.01]	***
Male	-1.88<br>[-2.83 -0.93]	***
Fulltime retired	-2.40<br>[-3.75 -1.05]	***
Intercept	30.65<br>[28.47 32.82]	***
var(_cons)	18.09<br>[15.44 21.20]	
var(e)	4.09<br>[3.92 4.26]	***
rho	0.31<br>[0.29 0.34]	***
Number of observations	6474	

Results are presented as unstandardised regression coefficients and 95% confidence intervals. * p <.05 ** p < .01 *** p < .001

Results from the supplementary sensitivity analysis (fully adjusted)

S3: Results from model fitting step 1: determining the residual and random effects structure (supplementary sensitivity analysis)

Supplementary Table S3. Results from model fitting step 1 (sensitivity analysis)

	**Log-likelihood**	**df**	**AIC**	***P* Comparison against M1**	**P Comparison against M2**
Sleep duration					
M1a Initial Model	-13046.71	21	26135.43		
**M1b Initial Model + autoregressive (AR1) residual structure**	**-12817.09**	**22**	**25678.18**	**< .001**	
M1c Initial Model + + autoregressive (AR1) residual structure + Random slope for sleep duration deviation	-12816.63	23	25679.26		0.3373
Sleep efficiency					
M1a Initial Model	-13018.42	21	26078.85		
**M1b Initial Model + autoregressive (AR1) residual structure**	**-12792.4**	**22**	**25628.8**	**< .001**	
M1c Initial Model + autoregressive (AR1) residual structure + Random slope for sleep duration deviation	-12792.4	23	25630.79		0.9070
Sleep quality					
M1a Initial Model	-13327.9	21	26697.81		
**M1b Initial Model + autoregressive (AR1) residual structure**	**-13089.95**	**22**	**26223.9**	**< .001**	
M1c Initial Model + +autoregressive (AR1) residual structure+ Random slope for sleep duration deviation	Not converged				

Bold print depicts the baseline model used in step 2 of the model fitting

Table S4: Results from model fitting testing the interactions between age group and the sleep predictors. Selected models are marked in bold (supplementary sensitivity analysis)

	**Log-likelihood**	**df**	**AIC**	***P*** Comparison against Baseline Model
Sleep duration				
**Baseline model**	-12817.09	22	25678.18	
Interaction Model 1	-12815.87	24	25679.73	0.2939
Interaction Model 2	-12817.09	23	25680.18	0.9664
Quadratic model	-12814.81	24	25677.62	0.1024
Sleep efficiency				
**Baseline model**	-12792.4	22	25628.8	
Interaction Model 1	-12791.42	24	25630.84	0.3748
Interaction Model 2	-12791.67	23	25629.33	0.2251
Sleep quality				
**Baseline model**	-13089.95	22	26223.9	
Interaction Model 1	-13089.56	24	26227.13	0.6809
Interaction Model 2	-13089.87	23	26225.74	0.6879

ll=log likelihood; df=degrees of freedom; AIC=Akaike Information Criterion, *p* = p-value
Baseline models included age group, the between-person and within-person component of the respective sleep predictor, a random intercept and an autoregressive (AR1) residual structure. Interaction Model 1 included in addition interaction terms between age group and both the between and within-person component of the sleep predictor. Interaction Model 2 included in addition to the baseline model the interaction term between age group and the within-person component of the sleep predictor. The quadratic model for sleep duration included in addition to the baseline model quadratic terms for the between and within-person components of sleep duration as fixed effects. All models were adjusted for the covariates sex, fulltime retirement, study day and study day^2, subjective health rating, self-reported daytime napping, weekend/holiday, married/co-living with partner, alcohol use, nicotine use

S5a-c: Full model specifications including covariates (sensitivity analysis)

Table S5a: Multilevel mixed modelling examining the effect of prior night sleep duration (actigraphy-based) as predictor of next day average number of correct responses in the mDSST (Results from supplementary sensitivity analysis)

Older adult group vs young adult group	-3.30	***
	[-4.51 -2.08]	
Average sleep duration (h) (between person effect)	0.25	
	[-0.49 0.99]	
Sleep duration (h) deviation (within person effect)	0.11	***
	[0.06 0.16]	
Study day	0.36	***
	[0.31 0.40]	
Study day quadratic	-0.01	***
	[-0.01 -0.01]	
Male	-1.67	**
	[-2.68 -0.67]	
Fulltime retired	-2.54	***
	[-3.85 -1.22]	
Subjective health rating (reference very good)		
good	0.03	
	[-0.10 0.17]	
neither good nor bad	0.22	*
	[0.02 0.41]	
bad	0.16	
	[-0.12 0.44]	
very bad	0.07	
	[-0.72 0.85]	
Self-reported nap		
	-0.17	**
	[-0.31 -0.04]	
Weekend/holiday		
	-0.14	**
	[-0.25 -0.04]	
Married/co-habiting with partner		
	-0.46	
	[-1.52 0.60]	
Alcohol category (reference category 0)		
1	0.65	
	[-0.48 1.78]	
2	0.90	
	[-0.51 2.31]	
Nicotine user		
	0.30	
	[-1.02 1.63]	
Insomnia Severity Score	-0.05	
	[-0.16 0.05]	
Intercept	27.94	***
	[22.97 32.92]	
var(_cons)	17.06	
	[14.50 20.07]	
var(e)	3.75	***
	[3.58 3.91]	
rho	0.33	***
	[0.30 0.36]	
Number of observations	6027	

Results are presented as unstandardised regression coefficients and 95% confidence intervals. * p <.05 ** p < .01 *** p < .001

Table S5b: Multilevel mixed modelling examining the effect of prior night sleep efficiency (actigraphy-based) as predictor of next day average number of correct responses in the mDSST (Results from supplementary sensitivity analysis)

Older adult group vs young adult group	-3.34	***
	[-4.53 -2.15]	
Average sleep efficiency (%) (between person effect)	0.01	
	[-0.07 0.09]	
Sleep efficiency (%) deviation (within person effect)	0.00	
	[-0.01 0.01]	
Study day	0.36	***
	[0.31 0.40]	
Study day quadratic	-0.01	***
	[-0.01 -0.01]	
Male		
	-1.74	***
	[-2.72 -0.76]	
Fulltime retired		
	-2.51	***
	[-3.81 -1.21]	
subjective health rating (reference very good)		
good	0.03	
	[-0.11 0.16]	
neither good nor bad	0.20	*
	[0.00 0.40]	
bad	0.16	
	[-0.12 0.44]	
very bad	0.01	
	[-0.78 0.79]	
Self-reported nap		
	-0.20	**
	[-0.33 -0.07]	
Weekend/holiday		
	-0.12	*
	[-0.23 -0.02]	
Married/co-habiting with partner		
	-0.44	
	[-1.48 0.60]	
Alcohol category (reference category 0)		
1	0.65	
	[-0.47 1.76]	
2	0.87	
	[-0.53 2.28]	
Nicotine user		
	0.28	
	[-1.02 1.59]	
Insomnia Severity Score	-0.05	
	[-0.15 0.05]	
Intercept	28.67	***
	[22.43 34.91]	
var(_cons)	16.53	
	[14.08 19.40]	
var(e)	3.75	***
	[3.58 3.91]	
rho	0.33	***
	[0.30 0.36]	
Number of observations	6010	

Results are presented as unstandardised regression coefficients and 95% confidence intervals. * *p* <.05 ** *p* < .01 *** *p* < .001

Table S5c: Multilevel mixed modelling examining the effect of prior night sleep quality as predictor of next day average number of correct responses in the mDSST (Results from supplementary sensitivity analysis)

Older adult group vs young adult group	-3.16	***
	[-4.40 -1.91]	
Average sleep quality index (between person effect)	-0.48	
	[-1.79 0.84]	
Sleep quality index deviation (within person effect)	-0.09	*
	[-0.16 -0.02]	
Study day	0.36	***
	[0.31 0.40]	
Study day quadratic	-0.01	***
	[-0.01 -0.01]	
Male		
	-1.92	***
	[-2.91 -0.93]	
Fulltime retired		
	-2.55	***
	[-3.91 -1.18]	
Subjective health rating (reference very good)		
good	0.04	
	[-0.10 0.18]	
neither good nor bad	0.23	*
	[0.03 0.43]	
bad	0.27	
	[-0.01 0.54]	
very bad	0.07	
	[-0.73 0.87]	
Self-reported nap		
	-0.26	***
	[-0.40 -0.13]	
Weekend/holiday		
	-0.13	*
	[-0.24 -0.03]	
Married/co-habiting with partner		
	-0.32	
	[-1.41 0.78]	
Alcohol category (reference category 0)		
1	0.51	
	[-0.65 1.67]	
2	0.88	
	[-0.57 2.33]	
Nicotine user		
	0.16	
	[-1.19 1.50]	
Insomnia Severity Score	-0.04	
	[-0.17 0.09]	
Intercept	30.29	***
	[27.92 32.66]	
var(_cons)	18.36	
	[15.62 21.57]	
var(e)	3.92	***
	[3.74 4.09]	
rho	0.34	***
	[0.31 0.37]	
Number of observations	6098	

Results are presented as unstandardised regression coefficients and 95% confidence intervals. * p <.05 ** p < .01 *** p < .001
